# An Approach to the Improvement of Graphene Production by Ultrasonic-Bath Treatment

**DOI:** 10.3390/nano15110817

**Published:** 2025-05-28

**Authors:** Bagila A. Baitimbetova, Danil W. Boukhvalov, Kostya A. Mit’, Tleuzhan S. Turmagambetov, Perizat Baitimbetova, Abay S. Serikkanov

**Affiliations:** 1 Department of Materials Science, Nanotechnology and Engineering Physics, Satbayev University, Satbayeva Str. 22, Almaty 050013, Kazakhstan; 2 Center for Two-Dimensional and Layered Materials, Pennsylvania State University, University Park, PA 16802, USA; 3 Institute of Physics and Technology LLP, Satbayev University, Ibragimova Str. 11, Almaty 050032, Kazakhstan; danil@njfu.edu.cn (D.W.B.); k.mit@sci.kz (K.A.M.); a.serikkanov@sci.kz (A.S.S.); 4 College of Science, Institute of Materials Physics and Chemistry, Nanjing Forestry University, Nanjing 210037, China; 5 Center Consulting, Tlendieva Str. 258b, Almaty 050060, Kazakhstan; ttleuzhan@yandex.ru; 6 Science and Innovation Park Abai Lab, Abai Kazakh National Pedagogical University, Dostyk Ave. 13, Almaty 050010, Kazakhstan; 47236@abaiuniversity.edu.kz; 7 National Academy of Sciences of the Republic of Kazakhstan under the President of the Republic of Kazakhstan, Shevchenko Str. 28, Almaty 050010, Kazakhstan

**Keywords:** graphene structure, exfoliate, ultrasonic treatment, graphene sheets

## Abstract

In this study, we report the synthesis of few-layer graphene via ultrasonic treatment of a graphite-benzene solution at room temperature. Raman spectroscopy revealed a significant reduction in the intensity ratio of the G and 2D peaks for samples subjected to 20 min of treatment, indicating a decrease in defect density and oxidation. Prolonged treatment times led to fragmentation of the graphene sheets, which facilitated restacking, as evidenced by Raman spectroscopy and microscopy. FTIR analysis confirmed the complete removal of the solvent from the extracted and dried graphene. Additionally, electron paramagnetic resonance (EPR) measurements indicated the presence of carbon-based magnetism in the synthesized samples, suggesting potential applications in spintronic devices. Our findings highlight the effectiveness of ultrasonic treatment for producing high-quality few-layer graphene with desirable structural and magnetic properties.

## 1. Introduction

One of the key challenges is the development of methods to produce graphene in quantities and quality that meet the demands of both research and industry. Therefore, the quality of graphene production is critical for its use in various applications, as highlighted in recent studies [1,2,3]. According to the commonly accepted definition, a stack of graphene crystals with more than ten layers at room temperature is considered bulk graphite [1].

For graphite, surface energy is the energy per unit area needed to overcome the van der Waals forces and separate two layers. The treatment of pure graphite with an organic reagent enhances the delamination effect of weak interlayer bonds. External energy is required to exfoliate the individual graphite layers. This energy is most often provided by the solution’s ultrasonic treatment. Over the past decade, the use of ultrasound to produce high-quality graphene from graphite dispersed in a liquid medium has gained significant attention due to its simplicity, low cost, and scalability potential [4]. Unlike chemical methods, such as oxidation followed by exfoliation and subsequent reduction, ultrasound liquid phase exfoliation (LPE) preserves the crystal structure of pristine graphene, which is essential for applications requiring high conductivity and low defect density [5,6,7].

Producing graphene through ultrasonic-assisted LPE of graphite flakes starts with selecting a suitable liquid phase where the graphite will be dispersed. Applying ultrasound generates cavitation microbubbles that create high-velocity microjets and shock waves, leading to regular and shear forces that break apart the graphite flakes and exfoliate individual graphene sheets [8]. Choosing the proper liquid phase is crucial for successful exfoliation, as an effective solvent helps counteract the van der Waals forces and stabilize the interactions between the π-orbitals of the graphene nanoplates, preventing them from restacking [9].

At the laboratory scale, two types of ultrasound devices are commonly used: ultrasonic tips (UST) and ultrasonic baths (USB). They differ in their operation. In UST, the acoustic intensity generated by the tip is directly transferred to the liquid phase with the graphite flakes. In contrast, USB uses a transducer, typically positioned at the bottom of the bath, to generate ultrasound waves. This bath is filled with water, and the liquid phase with graphite flakes is placed in a separate vessel, which is then submerged in the water. In this setup, the ultrasound waves travel through the water, pass through the vessel wall, and then reach the liquid phase with the graphite flakes. As a result, the acoustic intensity delivered by UST is higher than that of USB [10].

The power of the ultrasonic tip is consistently higher than that of the bath, making the tip sonicator significantly more efficient for dispersion than the bath sonicator under identical conditions [9]. In the case of composite fabrication, particularly for graphene dispersion, the detailed effects of variables such as ultrasonic power and duration, as well as solvent properties like viscosity, surface tension, and temperature, on graphene dispersion remain unclear [10,11,12,13]. Since the choice of experimental conditions is not optimal, the mechanical properties of the composites may be unsatisfactory due to the inhomogeneous graphene distribution. Previous research has shown that ultrasonic treatment can disperse graphene nanoplatelet agglomerates but may also lead to the fragmentation of graphene sheets [13]. Therefore, the size of LPE graphene flakes (in terms of lateral size or area) is typically small, usually around or less than 1 mm [11,12,13,14,15,16,17,18,19]. Despite this, for many prospective applications such as the improvement of construction materials durability [20,21,22], carbon fiber coating [23], or heating devices [24], graphene flakes do not need to be large, atomically thin, or perfectly crystalline [25].

Recent works report the production of graphene from graphite in N-methyl pyrrolidone (NMP), achieving graphene concentrations of 0.01 and 1.2 mg/mL after 0.5 and 270 h of ultrasonic bath (USB) sonication, respectively [6,9]. They also reported that ultrasonic tip (UST) sonication was ineffective for graphene exfoliation [6,9]. At the same time, A. A. Green and M.C. Hersam reported a graphene concentration of 0.09 mg/mL after 1 h using UST sonication [26]. Other researchers have proposed combining USB and UST treatments without clarifying the sequence of the methods used [17,27].

The work by J.T. Han et al. [8] demonstrates that graphite is almost entirely exfoliated into multilayer structures with approximately five layers in N,N-dimethylacetamide (DMA), g-butyrolactone (GBL), and 1,3-dimethyl-2-imidazolidinone (DMEU) and benzyl benzoate, but not in other solvents. This method leads to the appearance of a significant number of individual monolayers among the products. Furthermore, data from the study indicate that, for the best solvent (benzyl benzoate), 8.3% by mass of the original material remained after centrifugation. Thus, an organic hydrocarbon (benzene) is a reasonable choice as a solvent for the chemical destruction of the interlayer π–π bonds between layers in graphite plates. Thus, benzene is a suitable solvent characterized by surface tensions in the region of σ = 30 × 10^3^ H/m at room temperature.

The present work aimed to synthesize a graphene structure by ultrasonic bath treatment within 20 and 30 min of pure graphite with an organic reagent (benzene) at a fixed temperature to chemically break the weak interlayer bonds between graphite plates. Our study demonstrates that the production of graphene structures by ultrasonic processing is characterized by lower energy consumption and does not require significant time and resources compared to those reported in previous works. This approach provides high process efficiency while maintaining the quality of the fabricated materials.

## 2. Experimental

Structural properties were investigated using a TEM (transmission electron microscope, JEM-1011, Jeol Ltd., Tokyo, Japan). Accelerating voltage: 40 to 100 kV. Magnification: 800× to 600,000× in 30 steps. Low magnification from 50× to 1000× in 14 steps. Raman spectra were measured using an MT-MDT Integra Spectra at room temperature. The spectra were excited with a semiconductor laser with a wavelength of 473 nm (2.62 eV). A laser with a spot diameter of 2 μm on the sample was used to measure the spectra, which provided a sufficiently large irradiation area on the film surface. The accuracy of phonon frequencies was plus or minus 4 cm^−1^. The exposure time for measuring the spectra was 30 s. In addition, to avoid the impact of 1.5 milliwatt laser irradiation on the atomic structure of the films, the exposure was carried out in motion at a speed of about 10 μm per second. FTIR spectra of the samples were recorded as KBr tablets on a Nicolet iS5 infrared spectrometer (Thermo Scientific, Waltham, MA, USA) in a 4000~400 cm^−1^ frequency range [28].

The paramagnetic properties of the samples were measured by electron paramagnetic resonance (EPR) spectroscopy at room temperature using a JOEL JES-FA200 EPR setup in the 3 cm wavelength range. The sensitivity of the spectrometer was 5∙10^9^ spins/sample at 100 kHz modulation in a magnetic field. Mn^2+^ ions in MgO were used in the reference sample format. The signal from the studied and measured sample was recorded between the 3rd and 4th components of the six-line spectrum from Mn^2+^. A sample 3 mm × 5 mm was inserted into an ampoule made of special equipment glass, which does not give an electron paramagnetic resonance signal [29,30]. When the resonance conditions were obtained by varying the magnetic field within specific limits, an electron paramagnetic resonance signal was formed, which, after detection, was transmitted to print the spectrum of electron paramagnetic resonance to a printer. Electron paramagnetic resonance spectra are calibrated according to the well-known method described in Ref. [31].

## 3. Samples Fabrication

In our case, a circulation system was implemented to maintain a stable water temperature in the ultrasonic bath and prevent it from increasing, allowing water to enter and exit the bath simultaneously. The water supply was kept constant and uniform by installing a hose connected to a faucet or valve that regulated its flow. A stable water level was also maintained through the drainage channel, allowing water to be removed as new water came in, either using an overflow or an outlet at a certain height. In addition, a temperature sensor was used to monitor the temperature in real time and regulate the chilled water supply. This approach is the key difference from the works discussed in the introductory section, where warming the liquid in the bath was observed (see, for example, Ref. [9]).

**Figure 1:**
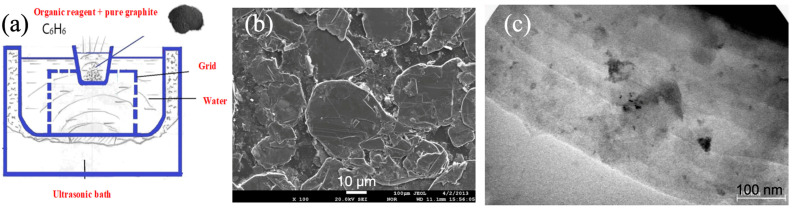
Scheme of the experiment on obtaining nanomaterials and graphene structures (**a**), SEM (**b**), and TEM (**c**) images of initial graphite powder.

The ultrasonic bath was used to exfoliate graphene in a graphite-benzene solution (see Figure 1a). The ultrasound generator irradiation frequency was 37 kHz, power was 150 W, and bath volume was −9.5 L. The characterization of graphite is reported in the Supplementary Information. After the ultrasonic bath treatment, the suspension was left alone to settle the coarse particles. The top liquid layer (supernatant) was then carefully drained to separate the liquid phase from the solid particles. The remaining precipitate containing exfoliated graphene structures was collected and dried. The resulting dry material was used for further analysis and characterization.

## 4. Results and Discussions

**Figure 2:**
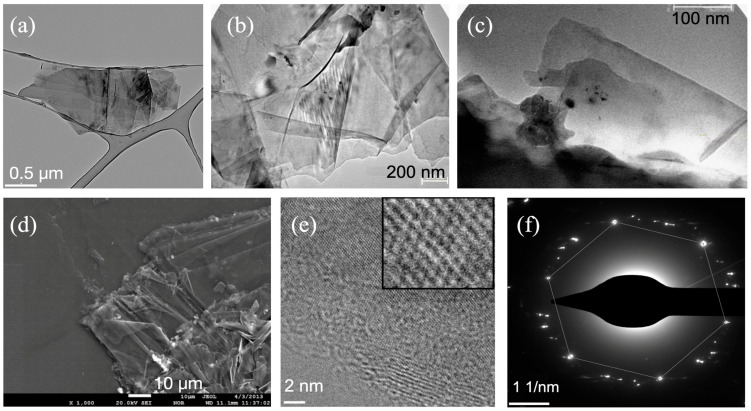
TEM images of the most representative graphene flakes fabricated for 20 min (**a**,**b**) and 30 min (**c**). SEM (**d**), HRTEM (**e**), and ED-TEM (**f**) images of the most representative graphene flakes fabricated for 30 min.

The TEM and SEM images (Figure 1b,c) indicate that the starting material was composed of flakes, aligning with earlier findings [9,18,19]. The ultrasonication significantly modified the surface morphology of graphite, leading to smaller and more uniform flake sizes as the sonication duration increased. TEM images of a few-layer graphene obtained after 20 min of ultrasonic treatment are shown in Figure 2a,b. These images evidence the efficiency of high-power ultrasonication in overcoming the van der Waals forces between adjacent graphite layers. In the image at high magnification (Figure 2b), separate, differently oriented planes and plates were visible, and some plates were curved, while others were slightly spaced relative to each other.

TEM images of the samples made after 30 min of treatment are shown in Figure 2c. As one can see, the morphology of these samples was similar to the initial graphite powder (see Figure 1b) and starkly different from the samples synthesized after 20 min. The TEM and SEM images of the samples synthesized in 30 min were also similar to the morphology of graphite powder (Figure 2c,d vs. Figure 1b,c). High-resolution TEM (HRTEM) images of this sample demonstrated rhombohedral patterns typical for graphite (see Figure 2e). Electron-diffraction TEM figures indicate graphitized carbon with a hexagonal structure under different imaging conditions (see Figure 2f) that corresponded with the formation of polycrystalline structures due to the fragmentation of graphite (Figure 2d vs. Figure 1b). However, the neat diffraction spots in three different orientations in Figure 2f can be attributed to the formation of some amount of multi-domain graphene.

**Figure 3:**
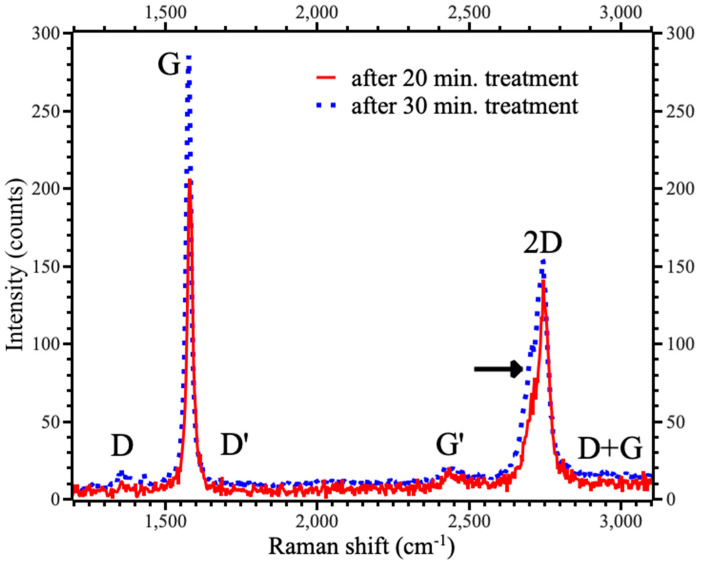
Raman spectra of samples fabricated at different times. The arrow indicates the split of 2D peak.

Raman spectroscopy is a powerful method to study graphite-to-graphene transition and evaluate the quality of the graphene [32]. The transition from graphite to graphene corresponds with a gradual decrease in the intensity (I) of the G peak and an increase in the intensity of the 2D peak [32]. These changes in the spectra are associated with a decrease in the contribution from interlayer bonds and an increase in the role of in-plane vibration in graphene sheets. Measured spectra of initial graphite powder were typical for graphite (see Figure S1). USB treatment led to a visible decrease in the intensity of the G peak in both samples. In the samples treated for 20 min, the I_G_/I_2D_ ratio was decreased to 1.46 (see Table 1). Thus, the formation of few-layer graphene with the number of layers above four can be proposed. The split of 2D peaks marked by the arrow in Figure 3 is also typical for four-layer graphene [32]. The presence of the G’ peak at about 2430~2440 cm^−1^ also evidences the low number of graphene layers in both samples [32]. On the contrary, the I_G_/I_2D_ ratio for the samples treated for 30 min was closer to the values reported for graphite [33]. However, several works reported a similar ratio for the few-layer graphenes [23]. Thus, we can propose restacking graphene flakes with an increase in the number of layers up to ten. Distinct appearance peaks in ultrasonically treated samples drastically differed from the broad peaks observed in graphene oxide samples, even after significant reduction [34]. Thus, we can claim the lack of oxidation of the samples under study. The absence of D′ (~1350 cm^−1^) and D + G (~3000 cm^−1^) bands in the Raman spectra demonstrates the lack of perforations in ultrasonically treated samples [35]. A minor D peak at 1356.6 cm^−1^ was observed in the spectrum of the samples fabricated for 30 min. The presence of the D peak and the broadening of the prominent peaks suggest an increase the quantity of defects with increasing treatment time [36]. This peak can also be associated with the formation of the grain boundaries between the domains (see Figure 2f and corresponding discussion), which formed as a result of the in-plane assembling of graphene sheets. Thus, based on the microscopy and Raman spectroscopy results, the treatment for 30 min at lower energy led mainly to the further fragmentation of the flakes without exfoliation of few-layered graphene. On the contrary, treatment for 20 min at higher energy led to the formation of high-quality, few-layer graphene. Thus, we excluded the samples fabricated within 30 min from further consideration. The possible cause of the restacking was the fragmentation of graphene with increased treatment time (see Figure 2d and corresponding discussion). This fragmentation should facilitate the restacking of graphene layers after finishing ultrasonic treatment.

**Figure 4:**
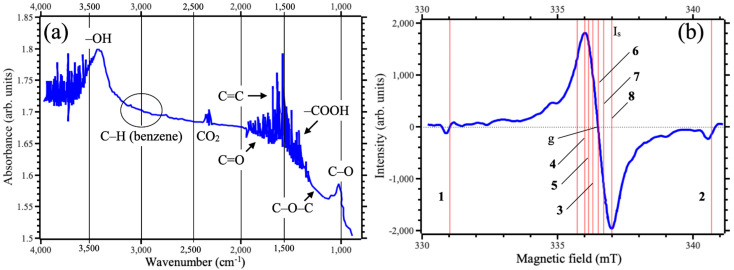
FTIR (**a**) and ESR (**b**) spectra for graphene obtained using 20 min of USB.

FTIR spectra were taken to check the removal of the solvent. Results of the measurements demonstrate the absence of the contributions from C–H stretching in the benzene in the area 3000~3030 cm^−1^ (Figure 4a). Thus, we can claim complete removal of benzene after centrifuging. Specific contribution from epoxy groups (C–O–C) with the frequency of about 1200 cm^−1^ [37] was also not detected. This observation is in agreement with the lack of oxidation detected in Raman spectra. The minor peak at about 2400 cm^−1^ was unambiguously associated with carbon dioxide [28,38,39]. This carbon dioxide can be formed by graphene sheet fragmentation in liquid media, as was observed during graphene oxide fabrication [40]. FTIR spectra also demonstrated a significant number of carboxyl groups (–COOH). The appearance of these groups was also associated with the fragmentation of graphene sheets in a liquid environment. The peak at about 3400 cm^−1^, unambiguously attributed to OH groups, can be associated with water molecules attached to hydrophilic groups on the edges. However, the peak at about 1060 cm^−1^ suggests that some OH groups belonged not to water but to C–OH edge terminations. A remarkable feature of the electrochemical exfoliation technique was that C=O, which is a reason for the presence of carbonyl groups, was not observed in the structure of graphene sheets [41]. This showed that electrochemical exfoliation was superior to other techniques.

Monovalent functional groups on zigzag edges of graphene are the source of magnetic moment in the absence of transitional metals [42]. EPR is a relatively simple and powerful technique to detect the magnetic impurities in graphene [29,30,43]. The results of EPR spectroscopy measurements for the studied samples are presented in Figure 4b and Table 2. As shown in Figure 4b, the EPR signal intensities were in the ratios of 3:5 and 8:4 for graphene synthesized via 20 min ultrasonic bath treatment. These results suggest that the formed thin graphene layers consisted of three distinct paramagnetic components. The first component corresponded to g = 2.00412~2.00421, which is characteristic of graphene and its derivatives [44]. The second component had a g-factor of 2.0030, which may be attributed to carbon nanostructures containing unpaired carbon bonds [45,46]. The third component showed a g-factor in the range of 2.00118 to 2.00198, depending on the orientation of the sample in the magnetic field. This variation indicates the presence of graphitic structures with varying degrees of crystallinity [29,46]. Well-established reference samples were used to ensure the reliability and calibration of the measurements. In particular, a sample of Mn^2+^ in MgO was employed due to its reproducible EPR signal. In the spectrometer setup, the Mn^2+^ in MgO reference sample was placed outside the resonator, in the scattered microwave field, resulting in opposite phase signals compared to the investigated sample. The reference Mn^2+^ signals were observed at g = 2.03256 and g = 1.98078, serving as calibration markers.

## 5. Conclusions

This study demonstrated the successful synthesis of few-layer graphene structures through the ultrasonic bath treatment of pure graphite in the presence of benzene as an organic reagent, conducted at a controlled liquid mix temperature. The continuous influx of chilled water effectively stabilized the bath’s temperature, thereby optimizing the synthesis process. The combination of the stable solvent temperature, chemical composition, and relatively short treatment time is the key difference from the previously reported works in this area.

Our approach contributes to a reduction in energy consumption for fabricating few-layer graphene while maintaining high quality. Raman and Fourier-transform infrared (FTIR) spectroscopy confirm that a treatment duration of 20 min resulted in the formation of high-quality few-layer graphene, devoid of oxidation or perforation, as further validated by microscopy.

Additionally, electron spin resonance (EPR) measurements indicated the presence of magnetic moments within the graphene, which we attribute to the presence of C–OH groups on the zigzag edges. This feature suggests potential applications of our synthesized graphene in spintronic devices.

FTIR spectra further demonstrated the complete removal of benzene from the isolated and dried graphene, indicating the effectiveness of our purification process. However, extending the treatment time to 30 min led to fragmentation of the graphene sheets, which facilitated restacking, a phenomenon confirmed by both Raman spectroscopy and microscopy. Therefore, selecting the appropriate treatment duration is critical for achieving graphene of desirable quality.

Moreover, the ultrasonic-assisted fragmentation of graphite in a liquid medium presents a promising avenue for increasing the yield of carbon quantum dots via a top-down approach. In summary, our findings underscore the importance of treatment conditions and duration in graphene synthesis, paving the way for future advancements in liquid phase exfoliation of various nanomaterials.

## Figures and Tables

**Table 1:** Positions and FWHD of G and D peaks and their intensities (I) ratio.

Time	G		2D		I_G_/I_2D_
	Position, cm^−1^	FWHD, cm^−1^	Position, cm^−1^	FWHD, cm^−1^	
20	1584.1	18.4	2774.8	53.1	1.46
30	1578.6	20.7	2742.0	71.8	1.85

**Table 2:** The parameters of the EPR spectra of graphene obtained by 20 min of USB.

Number	Field (mT)	G-Factor
**1**	331.031	2.03256
**2**	340.608	1.98078
**3**	336.421	2.00544
**4**	336.524	2.00393
**5**	336.675	2.00293
**6**	336.841	2.00293
**7**	337.031	2.0018
**8**	337.285	2.0003

## Data Availability

Data is contained within the article or Supplementary Materials.

## Supplementary Materials

The following supporting information can be downloaded at: https://www.mdpi.com/article/10.3390/nano15110817/s1, Figure S1. Elemental analysis of initial graphite powder. Figure S2. Raman spectra of the initial graphite powder.
