# Supplementary material for: An Approach to the Improvement of Graphene Production by Ultrasonic-Bath Treatment
Source: Nanomaterials (Basel). 2025 May 28;15(11):817. doi: 10.3390/nano15110817 (PMC12157132; doi:10.3390/nano15110817)
Supplement: Supplementary file 1 [file nanomaterials-15-00817-s001.zip › nanomaterials-3614158-supplementary.pdf]

nanomaterials

An approach to the Improvement of Graphene Production by Ultrasonic-Bath Treatment

Bagila A. Baitimbetova [1,2,*], Danil W. Boukhvalov [3,4], Kostya A. Mit' [3], Tleuzhan S. Turmagambetov T. S. [5], Perizat Baitimbetova [6] and Abay S. Serikkanov [3,7]

1 Department of Materials Science, Nanotechnology and Engineering Physics, Satbayev University, Satbayeva str. 22, Almaty 050013, Kazakhstan
2 Center for Two-Dimensional and Layered Materials, Pennsylvania State University, University Park, PA 16802, USA
3 Institute of Physics and Technology LLP, Satbayev University, Ibragimova str. 11, Almaty 050032, Kazakhstan;
4 College of Science, Institute of Materials Physics and Chemistry, Nanjing Forestry University, Nanjing 210037, China
5 Center Consulting, Tlendieva str. 258b, Almaty 050060, Kazakhstan; ttleuzhan@yandex.ru
6 Science and Innovation Park Abai Lab, Abai Kazakh National Pedagogical University, Dostyk ave. 13, Almaty 050010, Kazakhstan
7 National Academy of Sciences of the Republic of Kazakhstan under the President of the Republic of Kazakh-stan, Shevchenko str. 28, Almaty 050010, Kazakhstan
* Correspondence: baitim@physics.kz

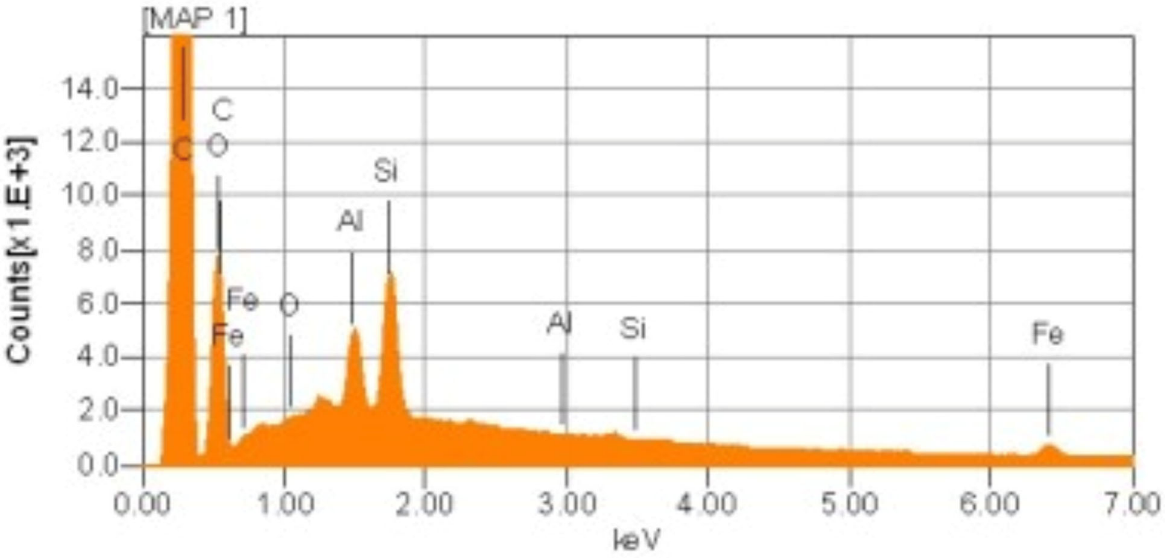

Figure S1. Elemental analysis of initial graphite powder.

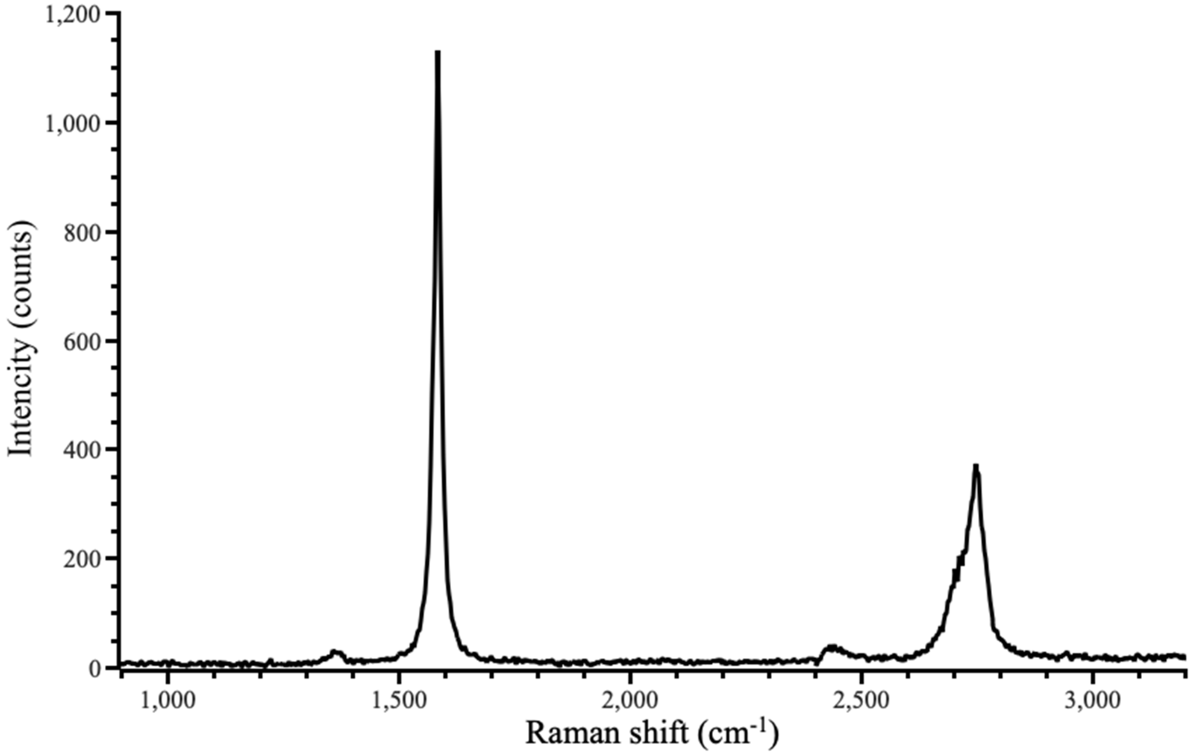

Figure S2. Raman spectra of initial graphite powder.
